# High-quality Fluorescence Imaging of the Human Acrosyringium Using a Transparency: Enhancing Technique and an Improved, Fluorescent Solvatochromic Pyrene Probe

**DOI:** 10.1267/ahc.20-00020

**Published:** 2020-11-21

**Authors:** Masamoto Murakami, Ryosuke Kawakami, Yosuke Niko, Teruko Tsuda, Hideki Mori, Kazuki Yatsuzuka, Takeshi Imamura, Koji Sayama

**Affiliations:** 1 Department of Dermatology, Ehime University Graduate School of Medicine, Ehime, Japan; 2 Department of Molecular Medicine for Pathogenesis, Ehime University Graduate School of Medicine, Ehime, Japan; 3 Research and Education Faculty, Multidisciplinary Science Cluster, Interdisciplinary Science Unit, Kochi University, Kochi, Japan

**Keywords:** human skin, sweat gland, two-photon excitation microscopy, solvatochromic pyrene probe, transparency-enhancing technology

## Abstract

Two-photon, excitation fluorescent microscopy featuring autofluorescence or immunofluorescence, combined with optical clearance using a transparency-enhancing technique, allows deep imaging of three-dimensional (3D) skin structures. However, it remains difficult to obtain high-quality images of individual cells or 3D structures. We combined a new dye with a transparency-enhancing technology and performed high-quality structural analysis of human epidermal structures, especially the acrosyringium. Human fingertip skin samples were collected, formalin-fixed, embedded in both frozen and paraffin blocks, sliced, stained with propidium iodide, optically cleared using a transparency-enhancing technique, and stained with a new fluorescent, solvatochromic pyrene probe. Microscopy revealed fine skin features and detailed epidermal structures including the stratum corneum (horny layer), keratinocytes, eccrine sweat glands, and peripheral nerves. Three-dimensional reconstruction of an entire acrosyringium was possible in one sample. This new fluorescence microscopy technique yields high-quality epidermal images and will aid in histopathological analyses of skin disorders.

## I Introduction

Human skin features an epidermis, dermis, and subcutaneous adipose tissue; the layers differ in thickness. Over many years, standard histological staining of skin sections, including hematoxylin-and-eosin (H&E) staining, has greatly advanced our understanding of normal and pathological morphologies; human skin biopsy is today a standard diagnostic procedure. Unfortunately, this approach does not allow three-dimensional (3D) tissue visualization in one slice; hundreds of slices must be Z-stacked by a computer. However, even excellent technicians find it difficult to obtain 100 continuous slices.

Fluorescence confocal laser scanning microscopy (CLSM) reliably and usefully reveals skin histopathologies, yielding high-quality signals from skin biopsies; 3D images are obtained via Z-stacking [12]. Two-photon excitation microscopy (TPM) is another 3D fluorescence imaging method employed by dermatologists [3, 13]. Compared to CLSM, which penetrates skin to only approximately 50–60 μm, TPM can image to a depth of about 300–600 μm (thus beyond the epidermis and superficial dermis) depending on the site, the excitation wavelength, and the fluorophore used [16]. Several skin researchers have improved resolution by combining TPM with autofluorescence or immunofluorescence techniques, yielding 3D skin structures [3, 11]. However, it remains challenging to obtain high-resolution 3D images of tissues or cells if a thick tissue section or block is observed via TPM in the absence of pretreatment using a transparency-enhancing technique. This novel technique is frequently used in work involving laboratory animals, especially to image brains [7, 14] and, when combined with laser scanning microscopy, yields Z-stacks running from the superficial to deep layers, enabling 3D reconstruction and analysis of morphological features. The various reagents/protocols are known by the terms BABB [4], 3DISCO [5], SeeDB [6], CLARITY [2] and CUBIC [14]. However, some require toxic reagents, expensive devices, and the use of complicated procedures. Also, clearing may be slow. The new ilLUmination of Cleared organs to IDentify target molecules (LUCID) method renders tissues transparent in a rapid and simple manner using non-toxic reagents [7]. Although dyeing and clearing may require 5–7 days, less time is required if the specimen is small and thin. The optimal transparency-enhancing technology depends on the samples to be studied and the experimental protocols.

Solvatochromic fluorescent dyes are known to sense the biomolecular organization of living systems by varying their fluorescence color in response to local polarity (lipophilicity). Most such dyes are not very bright and photostable, and exhibit limited spectral ranges and poor sensitivity to polarity. On the other hand, a push-pull structured pyrene probe was recently found to exhibit excellent brightness, photostability, and sensitivity to membrane lipid order/disorder. Such a pyrene probe realized high-contrast polarity mapping of organelles in living cells via two-color ratiometric detection and revealed that polarity changes in the following order: lipid droplets < plasma membranes < endoplasmic reticulum. The pyrene probe was also employed to study the organization of tissue lipophilicity in zebrafish embryo. Biomembranes, lipid droplets, cells, yolk, the extracellular space, and newly formed organs were associated with specific probe emission wavelengths [15]. Here, we combined use of the solvatochromic pyrene probe with a transparency-enhancing technology when examining skin tissues, especially epidermal structures of clinical importance such as the acrosyringium.

## II Materials and Methods

### Tissue sample preparation

Five fingertip skin samples from polydactyl patients were collected during operations. After removing the skin, tissue blocks (about 5 × 5 mm) were excised, fixed in freshly prepared 4% (v/v) paraformaldehyde in phosphate buffer (pH 7.4) for 24–48 hr at 4°C, and one block from each fingertip was frozen using a routine procedure and another was embedded in paraffin. Sections 5 μm in thickness were prepared for H&E staining, and sections 100 and 500 μm in thickness for fluorescence microscopy.

### Ethics

This study was conducted according to the principles of the Declaration of Helsinki. All subjects provided written informed consent and the work was approved by the Ethics Committee of Ehime University Graduate School of Medicine.

### Optical clearance using transparency-enhancing technology (OCTET) and solvatochromic pyrene probe (pyrene probe) staining

Samples were optically cleared using the LUCID method [7]. The pyrene probe has been previously described [15]. We prepared a 2-μM pyrene probe solution in LUCID; this served as the master mix solution (MMS) and was stored at room temperature. Tissue sections were first stained with propidium iodide (PI) in phosphate-buffered saline (PBS) overnight at room temperature. After several washes with PBS, the sections were immersed in the MMS for 76 hr at room temperature.

### Image acquisition

Tissue sections (of thickness 5, 100, and 500 μm) were subjected to light and fluorescence microscopy (BZ-X800, Keyence Corp., IL, USA). Also, images were obtained using an upright, confocal laser scanning microscope (A1R MP+, Nikon and MaiTai DS eHP, Spectra Physics, CA) fitted with a 25× water-immersion objective lens (Apo LWD 25×NA 1.10). The excitation laser frequency was 960 nm for all TPM imaging. To acquire second harmonic generation (SHG) signals, we divided the signals at the 495-nm point using a dichroic mirror. Pyrene probe and PI fluorescence signals were detected at 500–550 nm and 563–593 via a GaAsP-type photomultiplier module. For CLSM, the laser excitation wavelength was initially 403 nm and fluorescence signals from pyrene probe were acquired at 500–550 nm. Then, the excitation wavelength was shifted to 561 nm and fluorescence signals were acquired from 570 to 620 nm in Z-stack sequences (step size 5 μm), from the deepest level to the surface, corresponding to an area of 513 × 513 μm^2^ (1,024 × 1,024 pixels, 0.5 μm/pixel).

### Image analysis

Images were filtered and subjected to advanced denoising using Nikon NIS-Elements ver. 5.21 software. The reconstructed 3D image stacks were subjected to median 3 × 3 filtering. All samples were evaluated in the vertical axis and throughout the entire depths of the sections or blocks. Images of all vertical sections were stacked and saved as a single file in tag image format. The files were analyzed using NIS-Elements Advanced Research ver. 5.21 software (Nikon, Tokyo).

## III Results

Our new approach allowed us to observe (*in situ*) the various layers and compartments of human skin, including the stratum corneum, epidermis, dermis, and skin appendages. We performed 3D reconstruction of an entire human skin biopsy, in addition to detailed 2D observation.

### Autofluorescence microscopy observations

**Fig. 1.**
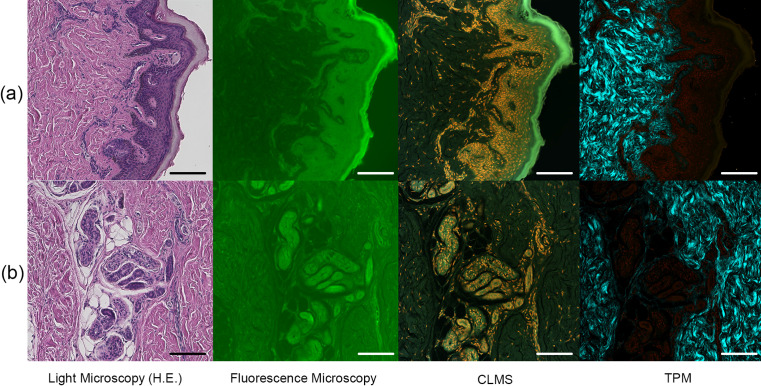
The epidermal and dermal sweat gland structures of skin tissue. A 5-μm slice was subjected to fluorescence microscopy, CLSM, and TPM after H&E staining. (**a**) The epidermis. (**b**) Sweat glands and ducts in the dermis. Bar = 100 μm.

After observation of the epidermis and dermal sweat gland structure via conventional H&E staining, tissues (5-μm-thick slices) were subjected to fluorescence microscopy (Fig. 1). This conventional microscopy revealed the silhouettes of the epidermis, dermis, and sweat glands. The resolution was less than that afforded by CLSM and TPM, which revealed the nuclei of various cells in addition to their silhouettes. The color combinations clearly revealed the epidermis, dermis, and dermal sweat glands. TPM also detected fine collagen fibers because the technique features SHG [16].

### Pyrene probe staining of human skin samples (frozen and paraffin blocks)

To view the tissue and cell structures at higher resolution, we prepared thick (100-μm) tissue sections. The OCTET/pyrene probe staining combination was evaluated using CLSM and TPM; both the resolution and focusing were poorer than obtained using the 5-μm slices (data not shown).

**Fig. 2.**
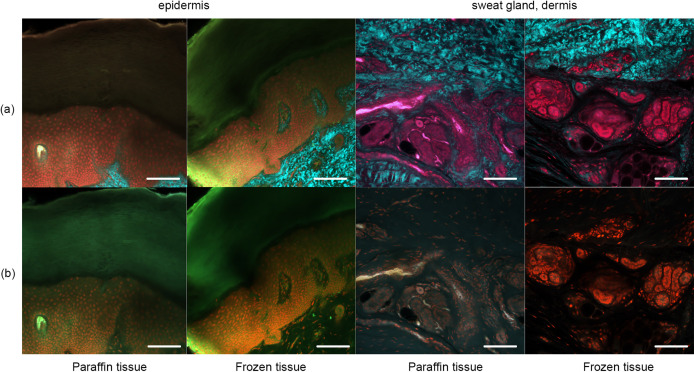
OCTET treatment of thick skin tissue. OCTET/PI-stained 100-μm-thick slices were prepared from both paraffin-embedded and frozen blocks and observed by (**a**) TPM and (**b**) CLSM. Bar = 100 μm.

Figure 2 shows the OCTET/PI results in the absence of pyrene probe staining. Epidermal structures were apparent in the 100-μm slices. The sweat gland components were as obvious as in the 5-μm slices (Fig. 1) when frozen and paraffin-embedded sections were viewed using CLSM and TPM. PI-stained nuclei were apparent, as in Figure 1.

**Fig. 3.**
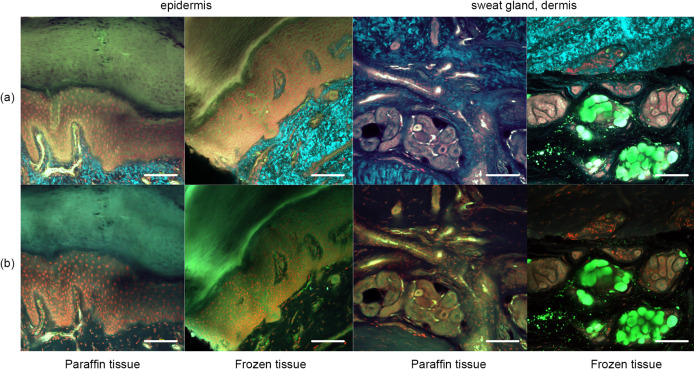
Pyrene probe staining after OCTET treatment of thick skin tissue. OCTET/pyrene probe/PI-stained 100-μm-thick slices were prepared from both paraffin-embedded and frozen blocks, and observed by (**a**) TPM and (**c**) CLSM. Bar = 100 μm.

Figure 3 shows the results after pyrene probe staining (thus using the OCTET/pyrene probe/PI protocol). Pyrene probe revealed the mesh-like patterns of the stratum corneum and epidermis, and the cell membranes. TPM of frozen tissue revealed a brick-like pattern of epidermal keratinocytes, which was also apparent in paraffin-embedded tissues observed using both TPM and CLSM. The epidermal acrosyringia, the focus of our interest, were well-revealed by TPM and CLSM after pyrene probe staining of both paraffin-embedded and frozen sections, as were the peripheral nerves of the upper dermis. In the deep dermis, the eccrine gland and duct structures were well-shown, and the inner lumina of glands and ducts also stained with pyrene probe in both paraffin-embedded and frozen sections. In the frozen sections only, TPM and CLSM revealed lipid droplets and the sebaceous glands. The signal quality from paraffin-embedded slices was similar to that from frozen slices, with the exception of the lipid droplets. Thus, all further experiments employed paraffin-embedded slices.

### Pyrene probe mediated deep imaging of skin tissue (paraffin-embedded blocks)

**Fig. 4.**
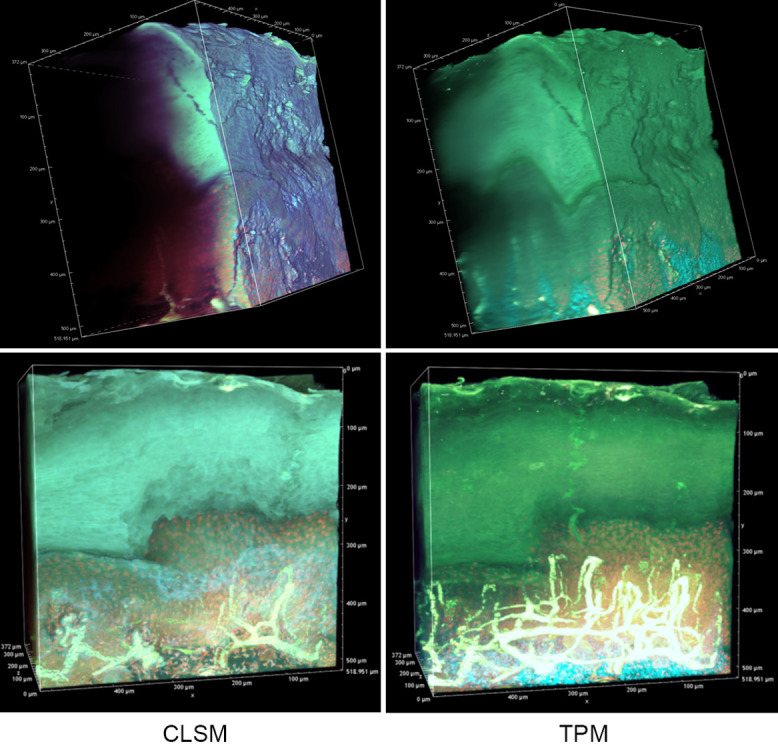
Deep imaging of OCTET/pyrene probe/PI-stained tissue. OCTET/pyrene probe/PI-stained 500-μm-thick slices were prepared from paraffin-embedded blocks and observed by CLSM (movie of Supplementary Fig. S1) and TPM (movie of Supplementary Fig. S2).

**Fig. 5.**
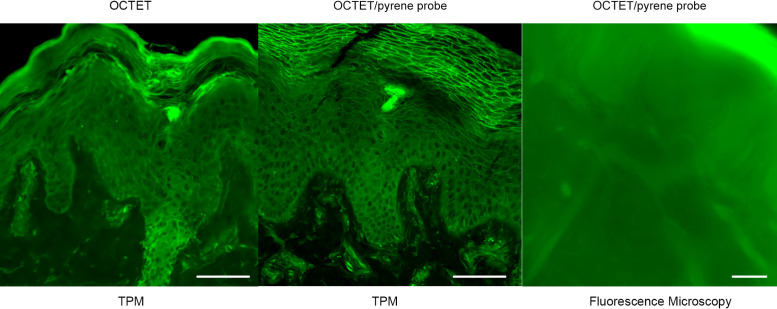
Deep imaging of epidermal structures stained with OCTET/pyrene probe. Slices 500-μm-thick were used to determine the fluorescence resolutions of TPM and a fluorescence microscope. Bar = 100 μm.

In an effort to reveal the entire, epidermal acrosyringial structure, we sought to deep-image 500-μm slices (Fig. 4, Supplemental Figs. S1, S2). As expected, CLSM penetrated to about 100–150 μm when creating 3D images with the aid of OCTET (Fig. 4, Supplemental Fig. S1) but TPM penetrated to about 500 μm, revealing tissue structures in exquisite detail (Fig. 4, Supplemental Fig. S2). We next attempted to visualize the fine structures of the epidermis, acrosyringia, and stratum corneum without PI staining (Fig. 5). Using 500-μm-thick slices, conventional fluorescence microscopy did not reveal fine details. However, OCTET/TPM revealed the stratum corneum, epidermal keratinocytes, and acrosyringial structures. Furthermore, pyrene probe staining increased the resolution, and also revealed the cell membranes (but not cytoplasm) (Fig. 5).

### Our new staining procedure (OCTET/pyrene probe) reveals the detailed, epithelial acrosyringial structure in paraffin-embedded blocks

**Fig. 6.**
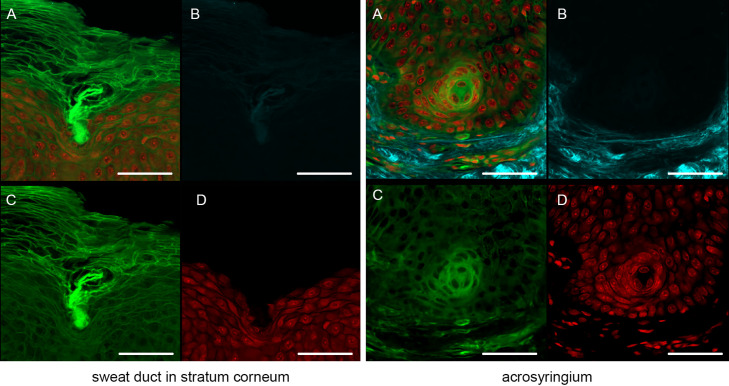
Sweat duct structures in the epidermis observed with the aid of OCTET/pyrene probe/PI and TPM. (**a**) Merged image, (**b**) SHG signal, (**c**) pyrene probe signal, (**d**) PI signal. Bar = 100 μm.

**Fig. 7.**
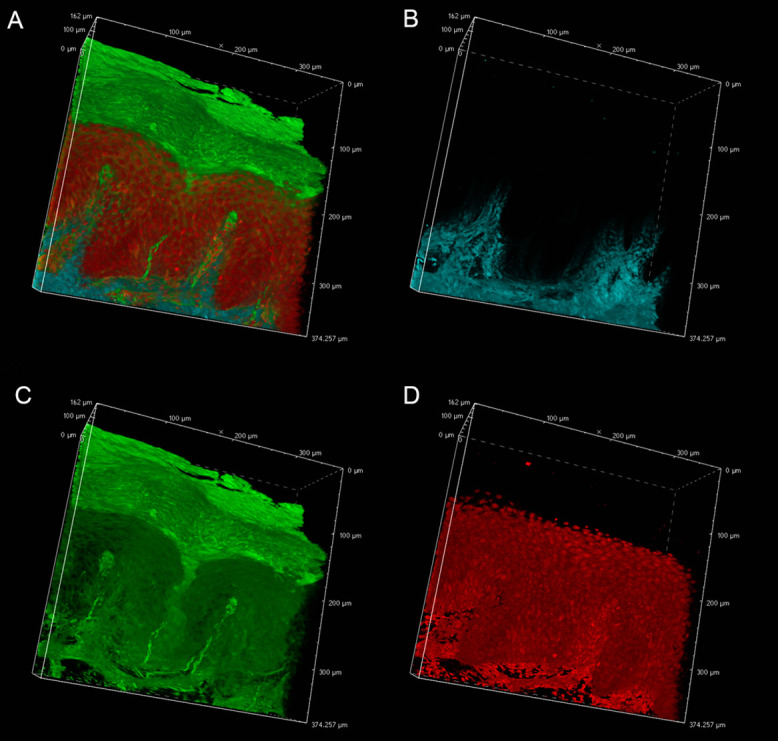
Three-dimensional imaging of epidermal structures. OCTET/pyrene probe-stained 500-μm-thick slices were used to determine the resolution of TPM fluorescence images. (**a**) Merged image, (**b**) SHG signal, (**c**) pyrene probe signal, (**d**) PI signal.

**Fig. 8.**
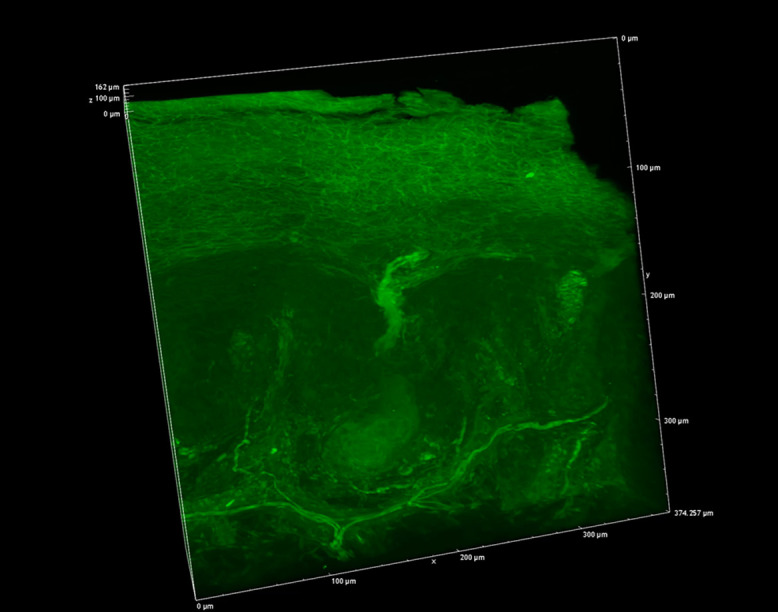
A 3D image of an entire epidermal acrosyringium (see the movie of Supplemental Fig. S3).

We next focused on the acrosyringia. Slices 500 μm in thickness were subjected to TPM after PI staining, OCTET, and pyrene probe staining (Figs. 6, 7). The green (pyrene probe), red (PI), and sky-blue (SHG) colors show the keratinocyte cell membranes, the nucleoli, and the elastic and collagen fibers respectively. A sweat duct in the stratum corneum (Fig. 6, left) and an acrosyringium near the basal layer of the epidermis (Fig. 6, right) were well-visualized. 3D images stained with pyrene probe, subjected to SHG, and stained with PI are shown in Figure 7. The merged image reveals the fine details of the epidermis and the stratum corneum (Fig. 7, upper left). Turning to the acrosyringium, the merged image of Figure 7 was excessively complex. Hence, we visualized the acrosyringium using only the pyrene probe signal (OCTET/pyrene probe, Fig. 8 and Supplemental Fig. S3). The entire acrosyringium was clearly observed, including the funnel-like structure on the epidermal surface (where the eccrine sweat duct enters the epithelial mass, and an asymmetrical spiral with two whorls on the upper two-thirds of the acrosyringium (the second whorl is wider than the first).

## IV Discussion

This is the first report to describe OCTET/pyrene probe staining of skin tissue (both frozen and paraffin-embedded slices) prior to CLSM and TPM. The OCTET/pyrene probe/TPM combination optimally deep-imaged *en bloc* specimens, yielding 3D acrosyringial reconstructions.

We have been working to elucidate the pathogenesis of palmoplantar pustulosis (PPP), a chronic pustular dermatitis involving the palms and soles. It shows several phenotypes such as vesicles, pustules, erythema, lichenification, and abnormal desquamation. Although it is one of Japan’s most common skin diseases, its pathomechanism is still unclear [10]. In 2010, we found and reported the relation between vesicle/pustule of PPP and acrosyringium [8]. Since then, acrosyringium of PPP lesion skin has been targeted to analyze its pathomechanism [9]. However, there is no established animal model of PPP, so that the study of PPP pathomechanism is not easy like psoriasis, which has many mimic animal models. The most informative source of PPP pathogenesis is still histopathological findings from PPP patients. However, it is challenging to see the whole PPP vesicle lesion at a glance in one picture because of the limitation of fundamental histopathological procedures. Hence, we needed a new method to see the PPP lesion as a three-dimensional picture, and we established an original method, high-quality fluorescence imaging of the human acrosyringium using a transparency-enhancing technique and an improved, fluorescent solvatochromic pyrene probe.

In healthy human skin, the intraepidermal portion of the eccrine sweat duct forms a highly symmetrical coil; this is the acrosyringium. The coil develops as the eccrine sweat duct attains the epidermis. Such coiling is not evident in any other adnexal skin structure (thus hair infundibula, or the apocrine or sebaceous ducts). Coiling is maintained despite constant cell renewal [1]. Several acrosyringial diagrams have been presented, usually drawn by reference to serially sectioned specimens. Employing 6–14 sections, 3D illustrations are successively drawn at magnifications of 200–400× and merged to show the coiled intra-epidermal portion of the sweat duct and the acrosyringium [1]. However, to the best of our knowledge, an entire acrosyringium has never been revealed in a single image. Conventional H&E-stained sections are usually 3–10 μm thick, and are examined via light or fluorescence microscopy. Many slides are required to obtain Z-axis data (depth); slide quality depends on the skill of the technician. CLSM yields high-resolution Z-axis data, but requires the use of a fluorescent dye for 3D reconstruction. However, as we have shown, CLSM penetrates to only about 100 μm; this is inadequate to reveal the entire acrosyringium [12, 16].

We present the entire 3D acrosyringeal structure in one image. We performed high-quality fluorescence imaging of the human epidermis (using OCTET) employing a new, fluorescent solvatochromic pyrene probe to reveal the fine detail of an entire acrosyringium and the associated epidermal structures via TPM. Solvatochromic dyes sense and image the biomolecular organization of living systems by monitoring local polarity (lipophilicity). pyrene probe is superior to other solvatochromic dyes in terms of brightness, photostability, and sensitivity to environmental polarity [15]. The dye is ideal for staining the lipid components of skin tissue, detecting cell walls, peripheral nerve sheaths, and sebaceous glands. We used the OCTET/pyrene probe/PI protocol to observe both frozen and paraffin-embedded tissue blocks via CLSM and TPM. The cytoskeletal architectures were brighter and clearer than those revealed by OCTET/PI. OCTET/pyrene probe/PI also yielded high-quality images of epidermal keratinocytes, the stratum corneum, and peripheral nerves. However, lipids in sebaceous glands were detected only in frozen tissue samples; the lipids were removed by xylene/alcohol during the preparation of paraffin-embedded blocks, but we have observed sweat glands in many archival samples, including histopathological specimens.

The staining procedure is elementary compared to H&E and immunofluorescence staining; thick tissue samples are placed in the MMS for several days. No special equipment is required prior to TPM. Staining reveals the cytoskeleton, not the cytoplasm. A combination of dyes that stain both compartments may be useful; we are currently optimizing that combination.

Pyrene probe staining using a transparency-enhancing technique, followed by TPM, afforded excellent views of the 3D structures of skin appendages and neural components. The combination of such staining with OCTET and TPM opens new applications in dermatology and dermatological research by allowing 3D visualization and analysis of the sweat glands of human skin.

## V Conflicts of Interest

All authors declare that they have no competing interests.

## VI Acknowledgments

The authors thank Dr. Tasuku Nishihara, Department of Anesthesia and Perioperative Medicine, Ehime University Graduate School of Medicine, Ehime, Japan, for his technical support. This work was supported by JSPS KAKENHI Grants 20K08691 and 18K09485, and a Grant-in-Aid for Scientific Research on Innovative Areal Platforms for Advanced Technologies and Research Resources from the “Advanced Bioimaging Support” program.

## VII Abbreviations

CLSM, confocal laser microscopy; TPM, two-photon excitation microscopy; SHG, second-generation harmonic; H&E, hematoxylin-and-eosin; pyrene probe, fluorescent solvatochromic pyrene probe; OCTET, optical clearance using transparency-enhancing technology.
